# Water lily pond: a multiomics database for water lilies

**DOI:** 10.1093/hr/uhaf076

**Published:** 2025-03-11

**Authors:** Chengjun Zhao, Ji Zhang, Yayu Chen, Lishuang Yang, Hongliang Chen, Yufan Liang, Wenquan Wang, Shuang He, Yunqing Luo, Junyu Zhang, Hongbin Zhang, Shuting Yang, Guilian Guo, Wenbai Dai, Zhijuan Yang, Junhao Chen, Yuhan Zhou, Wasi Ullah Khan, Guanhua Liu, Yifan Jiang, Tianlong Zhu, Yingchun Xu, Pedro García-Caparros, Yves Van de Peer, Jia-yu Xue, Chengjie Chen, Liangsheng Zhang, Fei Chen

**Affiliations:** National Key Laboratory for Tropical Crop Breeding, College of breeding and multiplication, Sanya Institute of Breeding and Multiplication, Hainan University, Sanya 572025, China; College of Tropical Agriculture and Forestry, Hainan University, Danzhou 571700, China; National Key Laboratory for Tropical Crop Breeding, College of breeding and multiplication, Sanya Institute of Breeding and Multiplication, Hainan University, Sanya 572025, China; College of Tropical Agriculture and Forestry, Hainan University, Danzhou 571700, China; National Key Laboratory for Tropical Crop Breeding, College of breeding and multiplication, Sanya Institute of Breeding and Multiplication, Hainan University, Sanya 572025, China; College of Tropical Agriculture and Forestry, Hainan University, Danzhou 571700, China; National Key Laboratory for Tropical Crop Breeding, College of breeding and multiplication, Sanya Institute of Breeding and Multiplication, Hainan University, Sanya 572025, China; College of Tropical Agriculture and Forestry, Hainan University, Danzhou 571700, China; National Key Laboratory for Tropical Crop Breeding, College of breeding and multiplication, Sanya Institute of Breeding and Multiplication, Hainan University, Sanya 572025, China; College of Tropical Agriculture and Forestry, Hainan University, Danzhou 571700, China; National Key Laboratory for Tropical Crop Breeding, College of breeding and multiplication, Sanya Institute of Breeding and Multiplication, Hainan University, Sanya 572025, China; College of Tropical Agriculture and Forestry, Hainan University, Danzhou 571700, China; National Key Laboratory for Tropical Crop Breeding, College of breeding and multiplication, Sanya Institute of Breeding and Multiplication, Hainan University, Sanya 572025, China; College of Tropical Agriculture and Forestry, Hainan University, Danzhou 571700, China; National Key Laboratory for Tropical Crop Breeding, College of breeding and multiplication, Sanya Institute of Breeding and Multiplication, Hainan University, Sanya 572025, China; College of Tropical Agriculture and Forestry, Hainan University, Danzhou 571700, China; National Key Laboratory for Tropical Crop Breeding, College of breeding and multiplication, Sanya Institute of Breeding and Multiplication, Hainan University, Sanya 572025, China; College of Tropical Agriculture and Forestry, Hainan University, Danzhou 571700, China; National Key Laboratory for Tropical Crop Breeding, College of breeding and multiplication, Sanya Institute of Breeding and Multiplication, Hainan University, Sanya 572025, China; College of Tropical Agriculture and Forestry, Hainan University, Danzhou 571700, China; National Key Laboratory for Tropical Crop Breeding, College of breeding and multiplication, Sanya Institute of Breeding and Multiplication, Hainan University, Sanya 572025, China; College of Tropical Agriculture and Forestry, Hainan University, Danzhou 571700, China; National Key Laboratory for Tropical Crop Breeding, College of breeding and multiplication, Sanya Institute of Breeding and Multiplication, Hainan University, Sanya 572025, China; College of Tropical Agriculture and Forestry, Hainan University, Danzhou 571700, China; National Key Laboratory for Tropical Crop Breeding, College of breeding and multiplication, Sanya Institute of Breeding and Multiplication, Hainan University, Sanya 572025, China; College of Tropical Agriculture and Forestry, Hainan University, Danzhou 571700, China; National Key Laboratory for Tropical Crop Breeding, College of breeding and multiplication, Sanya Institute of Breeding and Multiplication, Hainan University, Sanya 572025, China; College of Tropical Agriculture and Forestry, Hainan University, Danzhou 571700, China; National Key Laboratory for Tropical Crop Breeding, College of breeding and multiplication, Sanya Institute of Breeding and Multiplication, Hainan University, Sanya 572025, China; Department of Biology, Saint Louis University, St. Louis, MO 63103, USA; College of Agricultural & Biotechnology, Zhejiang University, Hangzhou 310085, China; National Key Laboratory for Tropical Crop Breeding, College of breeding and multiplication, Sanya Institute of Breeding and Multiplication, Hainan University, Sanya 572025, China; College of Tropical Agriculture and Forestry, Hainan University, Danzhou 571700, China; Tea Research Institute, Chinese Academy of Agricultural Sciences, Hangzhou 310008, China; College of Horticulture, Nanjing agricultural University, Nanjing 210095, China; Hainan Fodu Lianyuan Ecological Agriculture Co. Ltd., Haikou 570105, China; College of Horticulture, Nanjing agricultural University, Nanjing 210095, China; Agronomy Department of Superior School Engineering, University of Almería, 04120 Almeria, Spain; Department of Plant Biotechnology and Bioinformatics, Ghent University, Ghent 9052, Belgium; Center for Plant Systems Biology, VIB, Ghent 9052, Belgium; Department of Biochemistry, Genetics and Microbiology, Centre for Microbial Ecology and Genomics, University of Pretoria, Pretoria 0028, South Africa; College of Horticulture, Academy for Advanced Interdisciplinary Studies, Nanjing Agricultural University, Nanjing 210095, Nanjing, China; College of Horticulture, Nanjing agricultural University, Nanjing 210095, China; National Key Laboratory for Tropical Crop Breeding, Laboratory of Crop Gene Resources and Germplasm Enhancement in South China, Ministry of Agriculture and Rural Affairs, Key Laboratory of Tropical Crops Germplasm Resources Genetic Improvement and Innovation of Hainan Province, Tropical Crops Genetic Resources Institute, Chinese Academy of Tropical Agricultural Sciences, Haikou 571101, China; College of Agricultural & Biotechnology, Zhejiang University, Hangzhou 310085, China; National Key Laboratory for Tropical Crop Breeding, College of breeding and multiplication, Sanya Institute of Breeding and Multiplication, Hainan University, Sanya 572025, China; College of Tropical Agriculture and Forestry, Hainan University, Danzhou 571700, China

Dear Editor,

As one of the earliest diverging lineages of angiosperms, water lilies hold unique value for evolutionary studies [1]. Water lilies also hold significant cultural and economic value. For instance, the seeds of *Euryale ferox* are highly valued in traditional Chinese medicine as a starch-rich tonic with major health benefits [2]. Previously, we successfully sequenced and analyzed the first genome water lily (*Nymphaea colorata*), to provide multiple and valuable insights into the early evolution of angiosperms [3]. In recent years, multiomics data on water lilies have been steadily accumulating. Whole-genome sequencing [3–5], transcriptomics, and metabolomics analyses have provided a wealth of foundational resources to support water lily breeding research. However, these advances have also introduced new challenges—chiefly, how to effectively integrate and systematically analyze these diverse datasets in order to develop efficient genetic breeding strategies.

We started in 2018 to construct a database for water lilies, which we termed as Water Lily Pond (WLP). The WLP platform includes 11.14 Gb of genomic data from nine water lily species, encompassing a remarkable 409 321 genes. The transcriptomic data are equally extensive, with 1.2 Tb of *de novo* sequences and 1.235 Tb of reference-guided sequences. Rich annotation datasets—spanning gene families, transcription factors, KEGG pathways, GO terms, and SignalP predictions—comprise an extraordinary 3 034 356 entries. Moreover, the platform offers 141 932 expression profiles, 13 467 proteomic entries, 1841 metabolite data points, and phenotypic image data for 187 distinct species (Fig. 1A). Together, these resources establish WLP as an indispensable database for advancing water lily genomics and beyond. Website of the database: https://bioinformatics.hainanu.edu.cn/waterlily

**Figure 1:**
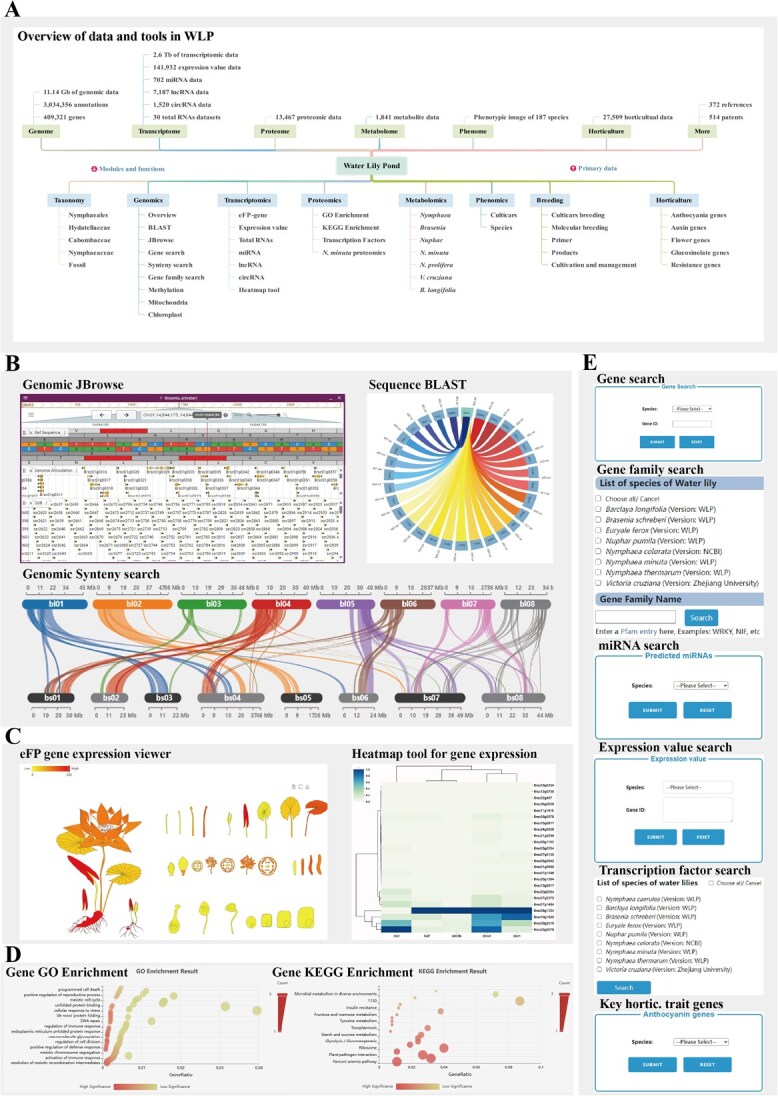
Highlighted data and tools integrated in WLP. (A) The datasets stored in WLP and the integrated tools in this database. (B) The Genomics module includes BLAST, JBrowse, and Synteny Search. (C) The transcriptomics module includes two important tools: eFP-gene tool, and Heatmap tool. (D) The proteomics module includes two critical tools: GO Enrichment, and KEGG Enrichment. (E) The main search tools, including gene search, gene family search, miRNA, expression value, transcription factor, and the Anthocyanin genes tool in the horticulture module.

WLP consists of seven primary modules each targeting a distinct area of research and community engagement: ‘Taxonomy’, ‘Genomics’, ‘Transcriptomics’, ‘Proteomics’, ‘Metabolomics’, ‘Phenomics’, ‘Breeding’ (Fig. 1A).

The module ‘Taxonomy’ compiles fundamental information on the order Nymphaeales, providing detailed biological descriptions. This includes comprehensive information on Nymphaeales, as well as the families Hydatellaceae, Cabombaceae, Nymphaeaceae, and fossils. For each genus within these families, an introductory content is provided.

The module ‘Genomics’ (Fig. 1B) integrates 10 water lily genomes and three main tools. Gene Search tool provides detailed information on gene sequences. BLAST tool suite not only provides the sequence alignment for genomes, genes, proteins, and also for the transcriptome sequences are also available. A Gene Family Search tool was provided for the easy access and comparison across water lilies. A chromosomal Synteny Search tool for checking syntenic relationships across water lilies are provided. A genome browser for visualizing genomic data and various annotations is integrated in WLP. Furthermore, WLP also provides specialized sections for methylation data under ‘Methylation’, mitochondrial genome information under ‘Mitochondria’, and chloroplast genome information under ‘Chloroplast’, providing a holistic perspective of genomic characteristics across multiple organelles.

WLP collected and sequenced the transcriptome data from 213 water lilies. The module ‘Transcriptomics’ (Fig. 1C) includes several tools for the visualization and the respective analysis of gene expression data. Notably, this module includes eFP-gene tool for visualizing expression patterns and the Heatmap tool for generating expression heatmaps. The module also features the expression value function for querying expression data across different conditions. Additionally, this module includes statistical analysis of miRNA, lncRNA, and circRNA data from the complete transcriptome of *N. minuta*, enabling in-depth exploration of noncoding RNA profiles within the species.

The module ‘Proteomics’ (Fig. 1D) provides users with a range of powerful analysis tools and informational resources, including two visualization tools for GO enrichment analysis (GO Enrichment) and KEGG enrichment analysis (KEGG Enrichment). These tools help users visually assess the enrichment of gene and protein functions, thereby revealing key pathways in biological processes. Additionally, the module includes proteomics data for *Nymphaea minuta* (*N. minuta* Proteomics), offering detailed data support for the study of protein characteristics and functions in this species.

The module ‘Metabolomics’ systematically compiles a rich dataset of metabolites, covering various species within the *Nymphaea*, *Brasenia*, and *Nuphar* genera, and further detailing important species such as *N. minuta*, *N. prolifera*, *Victoria cruziana*, and *Barclaya longifolia*. The module organizes metabolic data from different species into distinct functional categories, allowing efficient querying and navigation. Users can easily browse and analyze the unique metabolic profiles of each species, allowing comparative studies and the identification of species-specific metabolic features.

The module ‘Phenomics’ encompasses a wealth of visual resources, including flower and leaf phenotypic images for 180 different cultivars, showcasing the morphological diversity within these cultivars. Additionally, the module features a variety of tissue-specific phenotypic images from five wild species, covering different plant parts such as petals, leaves, and stems, providing a comprehensive display of the unique phenotypic characteristics of the original species across various tissue levels. This rich morphological information offers valuable insights into research and breeding applications supporting targeted breeding strategies.

The module ‘Breeding’ provides users with illustrated resources for common pests and diseases affecting water lilies and also offers comprehensive molecular breeding information to help in the identification and management of plant health issues. The module includes a molecular marker tool, Primer, for designing specific primers to more accurately select target genes in breeding programs.

The module ‘Horticulture’ systematically integrates detailed data on anthocyanin content, flowering time, floral scent composition, and flower color changes, enabling users to explore these traits in depth.

WLP also includes transcription factor data for nine species, providing critical resources for molecular mechanism studies. Additionally, the Library and Community modules aggregate a wide spectrum of academic resources and communication platforms, including literature, patents, open data, and detailed tutorials, thereby fostering knowledge exchange and supporting research collaboration.

In summary, WLP serves as the first and most comprehensive multiomics database. WLP provides researchers and breeders extensive genomic, proteomic, and transcriptomic data resources. The database not only offers first-hand genomic sequences, annotations, and various omics datasets, but also integrates multiple functional tools, allowing users to easily search and analyze specific genes or proteins, thereby facilitating cross-dimensional data analysis. Currently, the database achieves comprehensive annotation spanning genomics, proteomics, and transcriptomics, offering unique support for the exploration of gene functions, regulatory mechanisms, and protein interaction networks.

With the maturation and widespread adoption of third-generation sequencing technologies, such as PacBio, Oxford Nanopore, along with high-precision data processing tools, such as Hi-C, ATAC-seq, and scRNA-seq, the WLP platform will further offer more refined multidimensional omics data and innovative analytical tools. These new technologies support longer read lengths, lower error rates, and precise sequencing at the single-cell and spatial resolution levels, thereby facilitating research into genomic structural variation, epigenetic modifications, and cell-specific expression profiles. Moving forward, the WLP platform will continue to expand data types in areas such as genome assembly, transcriptome analysis, and epigenome annotation, while integrating efficient analysis tools and intelligent algorithms, enabling users to quickly complete the full analysis pipeline from data acquisition to insights. In addition, by incorporating high-quality visualization and interactive features, WLP will significantly enhance the depth and research value of multidimensional omics data, providing precise and comprehensive data support for plant biology and biodiversity research.

## Supplementary Material

Web_Material_uhaf076

## Data Availability

The data generated in this study have been released in the National Genomics Data Center, with GSA numbers: CRA019059 and CRA018961. The small RNA data can be accessed under GSA numbers: CRA019696 and CRA019738.
